# AI-enabled Cardiac Chambers Volumetry and Calcified Plaque Characterization in Coronary Artery Calcium (CAC) Scans (AI-CAC) Significantly Improves on Agatston CAC Score for Predicting All Cardiovascular Events: The Multi-Ethnic Study of Atherosclerosis

**DOI:** 10.21203/rs.3.rs-4433105/v1

**Published:** 2024-06-20

**Authors:** Morteza Naghavi, Anthony Reeves, Kyle Atlas, Chenyu Zhang, Thomas Atlas, Claudia Henschke, David Yankelevitz, Matthew Budoff, Dong Li, Sion Roy, Khurram Nasir, Jagat Narula, Ioannis Kakadiaris, Sabee Molloi, Zahi Fayad, David Maron, Michael McConnell, Kim Williams, Daniel Levy, Nathan Wong

**Affiliations:** HeartLung.AI; Cornell University; HeartLung.AI; HeartLung.AI; Tustin Teleradiology; Mount Sinai Hospital; Mount Sinai Hospital; The Lundquist Institute for Biomedical Innovation at Harbor UCLA Medical Center, Torrace, CA; The Lundquist Institute; The Lundquist Institute; Houston Methodist DeBakey Heart & Vascular Center; UTHealth Houston; The University of Texas Health Science Center at Houston; Department of Radiology, University of California Irvine; Icahn School of Medicine at Mount Sinai; Stanford University; Stanford; University of Louisville; National Heart Lung and Blood Institute; University of California at Irvine, Irvine

## Abstract

**Background:**

Coronary artery calcium (CAC) scans contain valuable information beyond the Agatston Score which is currently reported for predicting coronary heart disease (CHD) only. We examined whether new artificial intelligence (AI) algorithms applied to CAC scans may provide significant improvement in prediction of all cardiovascular disease (CVD) events in addition to CHD, including heart failure, atrial fibrillation, stroke, resuscitated cardiac arrest, and all CVD-related deaths.

**Methods:**

We applied AI-enabled automated cardiac chambers volumetry and automated calcified plaque characterization to CAC scans (AI-CAC) of 5830 individuals (52.2% women, age 61.7±10.2 years) without known CVD that were previously obtained for CAC scoring at the baseline examination of the Multi-Ethnic Study of Atherosclerosis (MESA). We used 15-year outcomes data and assessed discrimination using the time-dependent area under the curve (AUC) for AI-CAC versus the Agatston Score.

**Results:**

During 15 years of follow-up, 1773 CVD events accrued. The AUC at 1-, 5-, 10-, and 15-year follow up for AI-CAC vs Agatston Score was (0.784 vs 0.701), (0.771 vs. 0.709), (0.789 vs.0.712) and (0.816 vs. 0.729) (p<0.0001 for all), respectively. The category-free Net Reclassification Index of AI-CAC vs. Agatston Score at 1-, 5-, 10-, and 15-year follow up was 0.31, 0.24, 0.29 and 0.29 (p<.0001 for all), respectively. AI-CAC plaque characteristics including number, location, and density of plaque plus number of vessels significantly improved NRI for CAC 1–100 cohort vs. Agatston Score (0.342).

**Conclusion:**

In this multi-ethnic longitudinal population study, AI-CAC significantly and consistently improved the prediction of all CVD events over 15 years compared with the Agatston score.

## Introduction

Coronary artery calcium (CAC) scoring is the strongest predictor of risk for atherosclerotic cardiovascular disease (ASCVD) in asymptomatic individuals^1^. Although CAC scoring is used for prediction of overall ASCVD events, it is not used for prediction of other cardiovascular disease (CVD) events such as heart failure (HF) and atrial fibrillation (AF). Beyond risk factor assessment, screening tools for overall CVD event prediction are limited due to cost-effectiveness and feasibility barriers.

The usage of CAC scans has increased significantly since the ACC/AHA Guideline on the Management of Blood Cholesterol in 2018^2^ included CAC score in the algorithm for consideration of statin therapy, among those at borderline and intermediate risk for ASCVD. It is estimated that 45–50% of the US population aged 40–80 would fall in these groups defined as 5–20% risk of ASCVD events over 10 years^3,4^. The possibility of applying artificial intelligence (AI) to predict CVD has been previously published by some of our team members using the support vector machine algorithms in MESA^5^. We have sought to further enrich the value of CAC scans by applying AI that automatically measures all cardiac chamber volumes and left ventricular (LV) mass without using any contrast agent. For this manuscript, we refer to AI-enabled automated cardiac chambers volumetry from CAC scans as AI-CAC and the AI-CAC model incorporates Agatston CAC Score, LA, RV, LV volume and mass.

We have recently shown that AI-CAC volumetry alone enabled the prediction of HF in the Multi-Ethnic Study of Atherosclerosis (MESA)^6^. Additionally, we have demonstrated that AI-CAC LA volume alone improved on the predictive value of CHARGE-AF Risk Score and NT-proBNP for detection of individuals at high risk of AF^7,8^. Such an add-on measurement can offer valuable insights into a patient’s overall CVD risk beyond the CAC score. In this study of MESA participants, we compared the performance of AI-CAC over the traditional Agatston CAC Score for prediction of all CVD events.

## Methods

### Study population

MESA is a prospective, population-based, observational cohort study of 6,814 men and women without clinical CVD at the time of recruitment. Six field centers in the United States participated in the study: Baltimore, Maryland; Los Angeles, California; Chicago, Illinois; Forsyth County, North Carolina; New York City, New York; and St. Paul, Minnesota. As part of the initial evaluation (2000–2002), participants received a comprehensive medical history, clinic examination, and laboratory tests. Demographic information, medical history, and medication use at baseline were obtained by self-report. An ECG-gated non-contrast CT was performed at the baseline examination to measure CAC (see below).

### Outcomes

The primary outcome was a composite of all CVD events comprised of stroke, myocardial infarction, angina, HF, AF, resuscitated cardiac arrest, and all CVD-related deaths. Participants were contacted by telephone every 9–12 months during follow-up and asked to report all new CVD diagnoses. International Classification of Disease (ICD) codes were obtained. For participant reports of HF, coronary heart disease, stroke, and CVD mortality, detailed medical records were obtained, and diagnoses were adjudicated by the MESA Morbidity and Mortality Committee. Incident AF was identified by ICD codes 427.3x (version 9) or I48.x (version 10) from inpatient stays and, for participants enrolled in fee-for-service Medicare, from Medicare claims for outpatient and provider services. Hard CVD was defined as myocardial infarction, resuscitated cardiac arrest, stroke, CHD death, and stroke death. A detailed study design for MESA has been published elsewhere^9^. MESA participants have been followed since the year 2000. Incident AF has been identified through December 2018. 70 cases with AF diagnosed prior to MESA enrollment were removed from the analysis.

From the 6,814 MESA participants, we excluded 771 who did not consent to use of their data by commercial entities, leaving 6043 participants at baseline. Among the remaining participants, 125 participants with missing slices in CAC scans and 88 participants with missing event or time follow-up data were excluded, resulting in data from 5830 participants for final analysis. The 125 cases with missing slices in CAC scans were 49.8% male and 50.2% females with age 60.8±10.1. These errors were random, and our investigations did not reveal any association between cases with missing slices and any of the dependent or independent variables in our study.

### The AI tool for Automated Cardiac Chambers Volumetry

**Figure 1:**
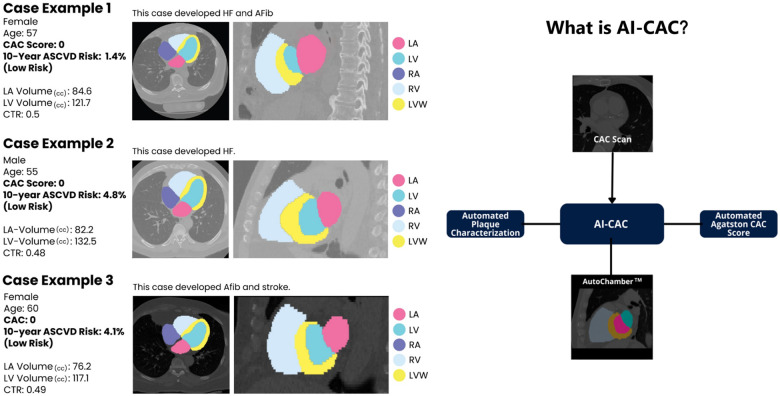
AI-CAC Component Diagram derived from a CAC Scan and Examples of AI-CAC Volumetry Detection of High-Risk Individuals with Enlarged Cardiac Chambers in Coronary Artery Calcium (CAC) Scans with CAC Score of zero. *MESA reported manually measured Agatston CAC Score was used in analyses for the AI-CAC model to show improvement over status quo.

The automated cardiac chambers volumetry tool in AI-CAC is referred to in this study is called AutoChamber^™^ (HeartLung.AI, Houston, TX), a deep learning model that used TotalSegmentator^10^ as the base input and was further developed to segment not only each of the four cardiac chambers; left atrium (LA), LV, right atrium (RA), and right ventricle (RV) but also several other components which are not presented here. Segmentations of cardiac chambers are shown in Figure 1. The base architecture of the TotalSegmentator model was trained on 1139 whole-body CT cases with 447 cases of coronary CT angiography (CCTA) independent from MESA using nnU-Net, a self-configuring method for deep learning-based biomedical image segmentation^11^. The initial input training data were matched non-contrast and contrast-enhanced ECG-gated cardiac CT scans with 1.5 mm slice thickness. Because the images were taken from the same patients in the same session, registration was done with good alignment. Following this transfer of segmentations, a nnU-Net deep learning tool was used for training the model. Additionally, iterative training was implemented whereby human supervisors corrected errors made by the model, and the corrected data were used to further train the model, leading to improved accuracy. To standardize the comparison in MESA, cardiac chambers were indexed by body surface area (BSA). AutoChamber was run on 6043 non-contrast CAC scans from participants that consented to commercial data usage out of the 6814 scans available in MESA exam 1. Expert rules built into the AI model excluded 125 cases due to missing slices in image reconstruction, which occurred with some of the electron-beam CT scanners used in MESA at baseline.

### Agatston CAC Score Measurement

Three study sites used cardiac-gated electron-beam CT scanners, whereas the other three sites used multidetector CT scanners. Each participant was scanned twice at baseline examination, with mean Agatston score used for analysis^12^. All scans were phantom-adjusted and read by two trained CT image analysts at a central MESA CT reading center, with high reproducibility and comparability between electron-beam CT and multidetector CT scanning^13,14^. Detailed information on CT scan methods and interpretation has been provided previously^13^.

CAC area and density were derived from total Agatston and volume scores, which were provided in the original MESA data set. The methods for this derivation are elsewhere^15^.

### AI-CAC Plaque Characterization beyond Agatston CAC Score

In addition to AI-CAC cardiac chambers volumetry, AI-CAC enables calcified plaque characterization that currently is not reported by the Agatston CAC Score. These characteristics include the number of plaques, number of vessels with plaques, plaque density and location. In this study, we have only used these characteristics for calcified plaques, however, efforts are underway to characterize non-calcified (soft plaques) in non-contrast CAC scans using AI-CAC.

### Statistical Analysis

We used SAS (SAS Institute Inc., Cary, NC) and R-4.3.3 software for statistical analyses. All values are reported as means ± SD except for CAC which did not show normal distribution and is presented as median with interquartile range (IQR). All tests of significance were two tailed, and significance was defined at Type I error (α) = 0.05 and Type II error (β) = 0.20. All analyses met the appropriate sample size and power considerations. Instances where these requirements were not met have been excluded and noted.

Survival analysis was performed using Cox proportional hazards regression. Discrimination was assessed using the time-dependent receiver operator characteristic (ROC) area under the curve (AUC)^16^ and Uno’s C-statistic^17^. The time-dependent AUC was calculated using the inverse probability of censoring weighting (IPCW) estimator without competing risks to determine discrimination at specific follow-up times. Significance in AUC difference was calculated based on the variance of the difference using the independent and identically distributed (iid)-representation of the estimator. Uno’s concordance was calculated to account for significant right censoring over 15 years of follow-up. Significance in concordance discrimination was determined using 2000 bootstrapped samples.

Category-free (continuous) net reclassification index (NRI) was calculated using the sum of the differences between the proportions of upward reclassifications and downward reclassifications events and non-events, respectively. P(up|event) and P(down|nonevent) form the positive components of the NRI in expression, while events that move down and nonevents that move up are mistakes introduced by the new marker. NRI was developed as a statistical measure to evaluate the improvement in risk prediction models when additional variables are incorporated into a base model^18^.

The AI-CAC model as presented is comprised of LA volume index, RV volume index, LV volume index, LV mass index, plaque characterization, and MESA-reported phantom-adjusted Agatston CAC score. Cardiac chamber volumetry was indexed by body surface area to standardize measurements. Agatston CAC score was natural logarithm-transformed (ln-transformed + 1) to improve the interpretability of hazards ratios and avoid undue influence of large values. All predictors were modeled continuously and exhibited a linear relationship with outcomes. The focus of this manuscript is comparing AI-CAC over Agatston CAC Score alone; therefore, no risk factors or other covariates were included in either model.

### Ethical Approval

As a longitudinal population-based study sponsored by the National Institute of Health (NIH), MESA has received proper ethical oversight. The MESA protocol was approved by the Institutional Review Board (IRB) of the 6 field centers (Columbia University IRB, Johns Hopkins Medicine IRB, Northwestern University IRB, UCLA Office of the Human Research Protection Program (OHRPP), University of Minnesota Human Research Protection Program, Wake Forest Baptist Health IRB) and the National Heart, Lung, and Blood Institute. Data from participants who did not consent to commercial use were removed from our study.

## Results

The mean (SD) age of our subjects was 62±10 years, 52% were female, 40% were non-Hispanic White, 26% non-Hispanic Black, 22% Hispanic, and 12.1% Chinese. Table 1 shows the baseline characteristics of MESA participants who experienced a CVD event versus those who did not over the 15 years of follow up, during which 1773 CVD events accrued. In univariate comparisons, participants experiencing CVD events were older, more likely male, and more likely non-Hispanic White. The cases that experienced a CVD event had higher cardiac chamber volumes for LA, LV, RA, and LV mass.

**Figure 2:**
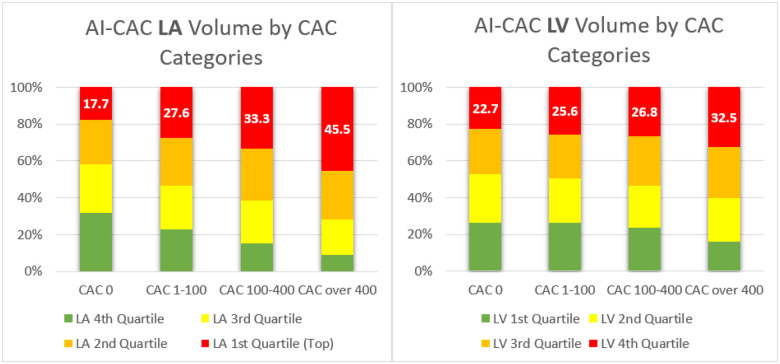
**a–b.** Quartiles of Artificial Intelligence-enabled Automated Cardiac Chambers Volumetry of CAC scans (AI-CAC) Left Atrial (LA) and Left Ventricle (LV) Volume by CAC Score Category.

Figure 1 shows examples of three participants with enlarged LA and LV volumes with CAC score 0 and low risk (<5%) ASCVD risk score who experienced CVD events. A significant number of low-risk participants with CAC 0 have enlarged cardiac chambers. With higher CAC score category, there was a higher proportion of patients with LA and LV volumes in the highest quartile (p-trend=0.0001). 17.7% of cases with CAC 0 who are considered low risk have enlarged LA volume that puts them at high risk for AF and stroke (Figure 2a). Similarly, 22.7% of cases with CAC 0 have enlarged LV volume that puts them at risk of HF (Figure 2b).

**Figure 3:**
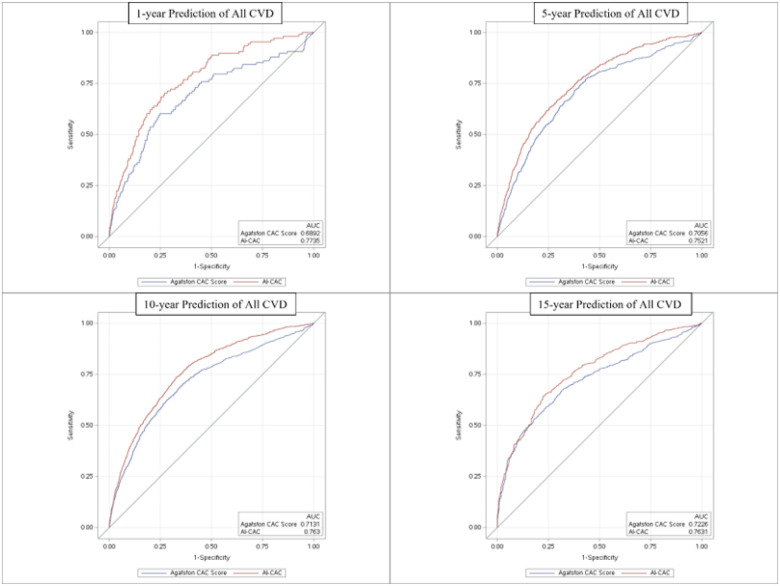
Time-Dependent Receiver Operating Characteristic (ROC) Area Under Curve (AUC) for cardiovascular events between Artificial Intelligence-enabled Automated Cardiac Chambers Volumetry to CAC scans (AI-CAC) vs Agatston Coronary Artery Calcium (CAC) Score over 1 to 15 years of follow-up. *AI-CAC: LA volume index, RV volume index, LV mass and volume index, Agatston CAC Score. †All Cardiovascular Events: stroke, myocardial infarction, angina, resuscitated cardiac arrest, all cardiovascular disease related deaths, heart failure, and atrial fibrillation.

The median C-statistic (95% CI) for all CVD events over 15 years for pooled sexes between AI-CAC vs. Agatston CAC score was 0.742 (CI: 0.723–0.761) vs. 0.709 (CI: 0.688–0.728) (p<0.0001). For females, the C-statistic between AI-CAC volumetry vs Agatston CAC Score was 0.751 (0.738–0.778) vs. 0.705 (0.683–0.720) (p<0.0001), respectively, and 0.701 (0.674–0.723) vs. 0.672 (0.651–0.693) (P=0.0012), respectively, for males. AI-CAC had significantly higher discrimination than Agatston CAC Score for CVD events prediction across 1-, 5-, 10-, and 15-year follow-up (Figure 3), including AF, HF, stroke, hard CVD, and All-Cause Mortality prediction (Table 2). Category-free NRI showed improvement across all follow-up periods for AF, HF, stroke, hard CVD, and All-Cause Mortality.

AI-CAC plaque characterization significantly improved CHD prediction in the CAC 1–100 cohort (Appendix A.2). The addition of AI-CAC RV volume, LV volume, and LV mass further improvement discrimination for CHD in this cohort. The AI-CAC composite model included RV volume, LV volume, LV mass, AI-CAC derived plaque characterization, and Agatston CAC Score. Over 5-and 10-year follow-up, the time-dependent AUC for the AI-CAC composite model vs. Agatston CAC Score was 0.654 vs. 0.557 (p<0.0001) and 0.688 vs. 0.556 (p<0.0001), respectively (Appendix A.1).

## Discussion

Our study primarily demonstrates the utility of applying AI to CAC scans to extract more actionable information than currently available which is the Agatston CAC score. We found that AI volumetry significantly improves upon traditional CAC scoring for the prediction of risk for total CVD events as well as the prediction of individual CVD events of HF, stroke, AF, and all-cause mortality in a large multiethnic cohort. The plaque characterization component of AI-CAC specifically improved on the predictive value of the Agatston score for CAC scores 1–100. Moreover, we show the value of this technique not only for longer-term event prediction (10–15 years), but also for nearer-term events (1 to 5-year follow-up). This is the first multi-ethnic outcome study of an easily implemented AI technology that can be applied to non-contrast CAC scans without additional radiation exposure to identify patients at risk of such events who would otherwise not be identified by traditional CAC scoring techniques. The potential utility of non-coronary findings in CAC scans has been reported previously using manual 2D measurements of LV^19,20,21,22^ and LA sizes^23,24,25,26^. Our study corroborates findings from the Heinz Nixdorf Recall Study and others, and further brings to light the value of non-coronary findings in CAC scans for a comprehensive CVD risk assessment beyond CHD^23,24,25,26,27^. Kizer et showed that LA size was an independent predictor of CVD events^28^. Mahabadi et al^24^ showed in the longitudinal Heinz Nixdorf Recall Study that two-dimensional LA size and epicardial adipose tissue from non-contrast CT were strongly associated with prevalent and incident AF and that LA size diminished the link of epicardial adipose tissue with AF, and was also associated with incident major CVD events independent of risk factors and CAC-score^25^.

Although there are multiple automated CAC scoring tools available, currently, there is no clinically available tool to clinicians for automated cardiac chambers volumetry in CAC scans that is validated against outcomes. Here, we provide evidence of the feasibility of using AI for automated 3D volumetry of cardiac chambers that takes on average 20 seconds. Currently such measurements are only possible on contrast-enhanced CT scans which require more radiation plus injection of an X-ray contrast agent that is burdensome^29^. In contrast, AI-CAC volumetry can be applied to any new or existing non-contrast CAC scan for automated cardiac chambers measurement. Standalone cardiac MRI and echocardiography are not comparable to our solution which is an opportunistic add-on to chest CT scans.

AI-CAC volumetry not only works on ECG-gated CAC scans but also non-gated lung CT scans^30^. Non-contrast chest CT scans are prime candidates for opportunistic AI-enabled cardiac chambers volumetry for identification of patients at increased risk for AF^7^ and HF. The AI approach can enable automatic screening of the over 10 million chest CT scans done each year in the US alone^31^. Such an AI tool can run in the background of radiology picture archiving and communication systems (PACS) and alert providers to cases with enlarged cardiac chambers. Unfortunately, many high-risk patients with enlarged cardiac chambers are currently undetected, and therefore untreated. Early detection of these cases can allow for close monitoring of progression to AF for stroke prevention and guideline-directed medical therapy for HF prevention. In our study, we have found the unadjusted correlations between Agatston CAC score and LA and LV volumes to be low (R=0.20 and R=0.10 respectively), hence a substantial portion of the population with enlarged LA and LV chambers are found in low-risk CAC categories. The combination of the automated cardiac chambers volumetry component of AI-CAC plus automated AI-CAC plaque characterization showed a greater incremental AUC value over the Agatston score versus each alone (Appendix A.1).

Finally, the lack of coverage for CAC scans by Medicare and healthcare insurance carriers has contributed to healthcare inequity in the US. Ikram and Williams^32^ have shown low-income people in Chicago area are less likely to get CAC scans comparted to people in higher income zip codes. We hope that by applying AI to CAC scans and providing incremental value, the payers will be more likely to cover CAC scans.

### Strengths and Limitations

Our study has several strengths and limitations. The multi-ethnic nature of MESA recruited from six field centers around the US provides for greater generalization of our findings than single-center studies. MESA included standardized methods of data collection, laboratory measurement, follow-up, as well as adjudication of CVD events.

One limitation is that the MESA Exam 1 baseline CT scans were performed between 2000 and 2002 using electron-beam computed tomography (EBCT) or earlier generation multidetector CT scanners, and current CAC scanning utilizes more advanced multidetector CT scanning. But since our AI training was done completely outside of MESA and used a modern multi-detector (256 slice) scanner, we do not anticipate this to affect the generalizability of our findings.

Since MESA does not distinguish between HF subtypes, (heart failure with reduced ejection fraction (HFrEF) vs. heart failure with preserved ejection fraction (HFpEF)) we were unable to compare the prediction of HF subtypes. However, in a preliminary study (abstract SCCT 2024) we obtained data from 75 patients who underwent both a cardiac CT scan and echocardiography at Harbor UCLA medical center. AI-CAC LV volume index (LVVI) defined as LV volume divided by BSA was able to distinguish HFrEF vs. HFpEF comparably to echocardiography LVVI (Appendix B).

## Conclusion

In this study, we presented AI-CAC data on cardiac chambers volumetry and calcified plaque characterization obtained from existing CAC scans in a large multi-ethnic prospective study and compared it to Agatston CAC Score alone for prediction of all cardiovascular events over 15 years. AI-CAC significantly improved upon the Agatston CAC score for all cardiovascular event prediction (including all CHD in CAC 1–100 cohort), as well as total mortality. Moreover, significant improvement in risk prediction and reclassification of events was not only seen for longer-term (e.g., 10-and 15-year) events, but also for nearer-term (e.g., 1-and 5-year) events, providing a useful means to help identify individuals at risk of near-term CVD events and death.
